# Phytoplankton biodiversity and the inverted paradox

**DOI:** 10.1038/s43705-021-00056-6

**Published:** 2021-10-06

**Authors:** Michael J. Behrenfeld, Robert O’Malley, Emmanuel Boss, Lee Karp-Boss, Christopher Mundt

**Affiliations:** 1 Department of Botany and Plant Pathology, Oregon State University, Corvallis, OR USA; 2 School of Marine Sciences, University of Maine, Orono, ME USA

**Keywords:** Biodiversity, Biogeography

## Abstract

Earth’s aquatic food webs are overwhelmingly supported by planktonic microalgae that live in the sunlit water column where only a minimum number of physical niches are readily identifiable. Despite this paucity of environmental differentiation, these “phytoplankton” populations exhibit a rich biodiversity, an observation not easily reconciled with broadly accepted rules of resource-based competitive exclusion. This conundrum is referred to as the “Paradox of the Plankton”. Consideration of physical distancing between nutrient depletion zones around individual phytoplankton, however, suggests a competition-neutral resource landscape. Application of neutral theory to the sheer number of phytoplankton in physically-mixed water masses yields a prediction of astronomical biodiversity, suggesting the inverted paradox: Why are there so few phytoplankton species? Here, we introduce a trophic constraint on phytoplankton that, when combined with stochastic principals of ecological drift, predicts only modest levels of diversity in an otherwise competition-neutral landscape. Our “trophic exclusion” principle predicts diversity to be independent of population size and yields a species richness across cell-size classes that is consistent with broad oceanographic survey observations.

## Introduction

Understanding processes governing biodiversity has been a long-standing pursuit that unites ecologists across all systems of study and is fundamental to evolutionary interpretations, assessments of ecosystem stability, and predictions of vulnerabilities. Natural selection through niche-specific competition is a common neo-Darwinian explanation for observed diversity, with a supposition that steady-state species richness increases with niche differentiation. Based on this perspective, Evelyn Hutchinson articulated the now-famous “Paradox of the Plankton”,

“*The problem that is presented by the phytoplankton is essentially how it is possible for a number of species to coexist in a relatively isotropic or unstructured environment all competing for the same sorts of materials. … According to the principle of competitive exclusion, we should expect that one species alone would outcompete all the others so that in a final equilibrium situation the assemblage would reduce to a population of a single species*” [1].

In other words, why are so many phytoplankton species found suspended in water columns that have so few readily-identifiable niches? Modern genomic technologies have only made this question more profound, as their application to broad geographic surveys has uncovered a far richer, often cryptic, level of diversity amongst the phytoplankton [2–4]. Resolving this mystery can proffer insights on biodiversity that transcend boundaries of aquatic and terrestrial ecology and can improve predictions of future ecosystem change and resilience.

For Hutchinson [1], competitive exclusion was at the heart of the phytoplankton paradox and entailed a “bottom-up” struggle between species for acquiring limiting resources. Accordingly, subsequent attempts to explain the paradox have tended to focus on this issue. For example, one element contributing to the unexpected diversity of phytoplankton is thought to be that nutrient-based species alliances, or co-dependencies, create a plethora of ecological niches within an otherwise homogeneous physical landscape [2, 5]. Environmental disturbance is another commonly proposed mechanism for maintaining diversity, whereby temporal changes in growth conditions prevent exclusion by shifting competitive advantages for resource acquisition amongst species [1, 6–8]. It is noteworthy that environmental disturbance disrupts ecological niches [9], so these two mechanisms for maintaining diversity can counteract each other. A radically different possibility is that phytoplankton biodiversity is simply not governed by resource-based competition.

At nearly all naturally occurring concentrations, the average body-length spacing between individual phytoplankton is such that nutrient depletion zones rarely overlap between neighboring cells, making direct competition unlikely [10, 11], and rapid resource recycling and physical mixing effectively erase any depletion-zone signatures of species-specific physiologies [12]. Behrenfeld et al. [12] described this natural condition of broad spatial distancing between individuals as a “competition-neutral resource landscape” and proposed competition-independent mechanisms governing phytoplankton size distributions and succession. With respect to biodiversity, neutral theory can provide a framework for understanding community composition in the absence of resource-based competitive exclusion. In neutral theory, individuals across all species are assumed equally susceptible to random deaths and abundance-dependent reproduction causes a stochastic walk, or “ecological drift”, that dictates biodiversity [13–15]. Neutral theory is often explored in studies of terrestrial communities, but can its application to the competition-neutral resource landscape of aquatic systems provide an explanation for the Paradox of the Plankton?

Here, we show that neutral theory places little constraint on phytoplankton biodiversity, but when combined with selective top-down ecological processes yields predictions of diversity compatible with observations. We then use this construct to evaluate how speciation rates and genome-size evolution structure the size distribution of phytoplankton diversity. Our theoretical expectations are then broadly compared to observations from the *Tara* Oceans expedition. Our findings give new insights on mechanisms governing biodiversity, identify a fundamental missing element in neutral theory, and provide an explanation for the Paradox of the Plankton.

## Results and discussion

### Inverted paradox

**Fig. 1:**
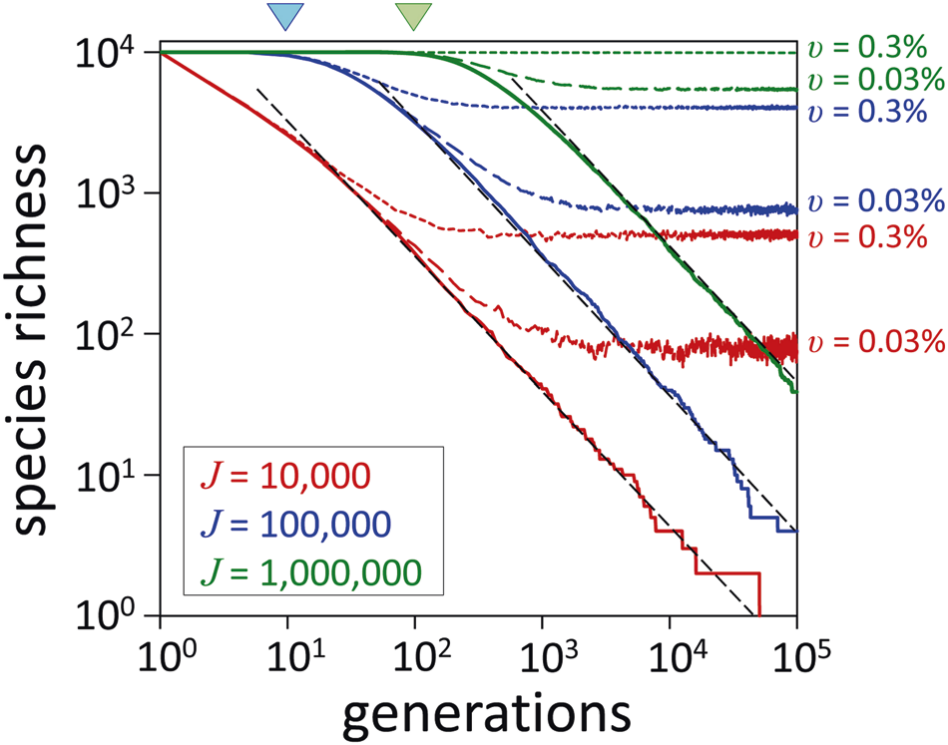
Phytoplankton biodiversity following purely stochastic processes. Red, blue, and green = phytoplankton populations (*J*) of 10,000, 100,000, and 1,000,000 individuals, respectively (Methods). Colored solid lines = species richness in the absence of immigration (*υ*). Colored dashed and dotted lines = species richness for *υ* values of 0.03% and 0.3% per generation. Black dashed line = mean rate of decline for the primary phase of stochastic exclusion (slope of this line is the same for all three populations). Blue and green downturned triangles = threshold for the two larger populations where diversity begins to decline rapidly because a sufficient number of species have been reduced to an abundance where extinction within a generation becomes likely.

Neutral theory can reproduce properties of terrestrial biodiversity observed at local (e.g., an island) or metacommunity (i.e., a set of interacting communities linked by dispersal of species) scales, particularly ranked species abundance curves (i.e., histograms of species abundance ordered along the x-axis from most to least common) [14]. Central to neutral theory is the interplay between ‘stochastic exclusion’ and either immigration or speciation. Stochastic exclusion is the reduction in biodiversity caused by random deaths and abundance-dependent replacement and, if not countered by other processes, ultimately leads to only a single remaining species [14]. Immigration of species into a local community or speciation within the metacommunity offset stochastic exclusion and maintain biodiversity [14]. This relationship is illustrated in Fig. 1 by simulated time-series of phytoplankton diversity for three populations at steady-state with 10,000, 100,000, and 1,000,000 total individuals and an initial condition of 10,000 species each (Fig. 1) (Methods). Subjection of these populations to 50% random mortality per generation and replacement in proportion to the relative abundance of remaining species results in an eventual rate of decrease in diversity that is equivalent across population sizes (Fig. 1; dashed black lines), eventually yielding the expected final equilibrium of a single species. When a small rate of immigration is added to this simulation (here, 0.03% or 0.3% per generation), complete stochastic exclusion is replaced by steady-state diversities that vary in direct proportion to population size and immigration rate (Fig. 1; colored dashed and dotted lines). Similar considerations led Hubbell [14] to earlier propose in his “Unified Neutral Theory” a fundamental biodiversity number, *θ*, controlling both species richness and relative abundance:

1 $$\theta \,=\, 2J\upsilon$$

where *J* is the total number of individuals in the community and *υ* the rate of immigration (local) or speciation (metacommunity).

In addition to illustrating the balance between stochastic exclusion and immigration into a local phytoplankton community, Fig. 1 shows that significant decreases in species richness only ensue after a subpopulation of species within a community has been sufficiently decimated in number that their remaining individuals might be lost through random mortality within a generation. In our simulations, this threshold is demarked by the downturn in species richness for the populations of 100,000 and 1,000,000 individuals (Fig. 1; blue and green triangles). The significance of stochastic exclusion is thus dependent on the relation between extant species number and size of the physically-homogenized community. With respect to the latter property, typical horizontal eddy diffusion values for the upper ocean are O(10^3^ m^2^ s^−1^), implying that the length scale for mixing in 1 day is O(1000 m). Typical number concentrations for phytoplankton of different species in the ocean range from <1 to over 10^5^ individuals per milliliter. At these concentrations, extension of our stochastic model results to the population encompassed within a water mass only 1 m deep by 1000 m wide yields an onset time for significant stochastic exclusion of 10^5^ to 10^10^ years, comparable to the age of life on Earth (Methods). Recognizing that the entire surface layer of the global ocean is homogenized on a time-scale of only O(1000 years), we can confidently conclude that the stochastic principals underlying neutral theory allow for an astronomically large phytoplankton biodiversity.

Traditional morphologically-based and modern genomics-based surveys yield estimates of planktonic diversity on the order of tens of thousands of phytoplankton species [2–4, 16, 17]. Such numbers pale in comparison to, for example, the hundreds of thousands of species of terrestrial plants [18]. Given the apparent competition-neutral resource landscape experienced by phytoplankton [10–12], the essentially unlimited potential for diversification afforded under neutral theory by the sheer number of phytoplankton in mixed water masses, and a geological history for speciation that far outdates that of terrestrial organisms, we propose that the Paradox of the Plankton is not “Why are there so many phytoplankton species”, but rather the inverse, ‘Why are there so few’?

### Trophic exclusion

One of the stark differences between terrestrial and aquatic ecosystems is that turnover of photosynthetic biomass is generally on the order of months to centuries for the former and only days for the latter [19, 20]. Picophytoplankton, for example, in the temporally-stable oligotrophic central ocean gyres have a community-averaged growth rate of roughly one division per day [21] and are consumed by small generalist grazers (and their nutrients subsequently recycled) at an equivalent rate. While the average physical distancing between picophytoplankton largely precludes overlap of nutrient depletion zones between cells [10, 11], physiological differences between species can allow some species to locally extract more limiting nutrient per day than others. Consequently, the potential exists for a distribution of growth rates across phytoplankton species within a given size class. If mortality is random among species (i.e., untargeted and equal to the average division rate of the ensemble picophytoplankton community), each successive “average generation” bestows a slight advantage for species with enhanced division rates and disadvantage for species with lower division rates. Accordingly, tight trophic coupling between the production of a phytoplankton community and its consumption can, in the high-turnover world of the plankton, selectively constrain species diversity in an otherwise competition-neutral resource landscape, a process we refer to here as “trophic exclusion”.

**Fig. 2:**
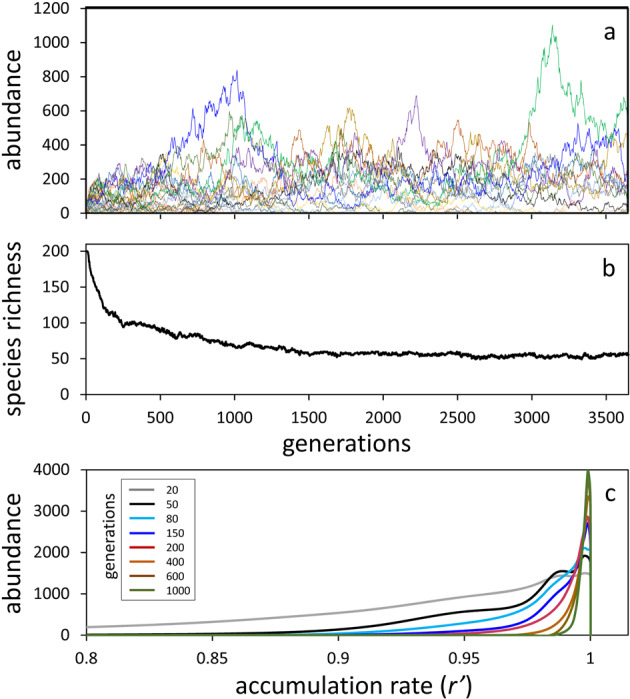
Phytoplankton biodiversity under directed ecological drift from trophic exclusion. **a** Abundance trajectories over 3650 generations for 50 (randomly selected) of the 200 model species, where division rates (*µ*) are predefined and total population size is 10,000 individuals (Methods). Short-term changes in the abundance of different species reflect stochastic drift, but both trophic exclusion and stochastic exclusion decrease total diversity as the number of modeled generations increases. **b** Total number of remaining unique species as a function of modeled generations. **c** Frequency histograms of species accumulation rates (*r*′) within the population as a function of modeled generations (colors defined in inset box). Smoothed curves are based on averages for three independent runs of the model. The value of *r*′ is determined by the balance between division and loss rates, which for these simulations is determined by assigned division rates (i.e., *r*′ = *µ*) but in nature can be equally impacted by life strategies that impact species-specific loss rates.

The influence of trophic exclusion on biodiversity can be illustrated by modifying our stochastic model (Fig. 1) such that 200 species are evenly distributed within a population of 10,000 individuals and each species is assigned one of ten fixed division rates ranging from 0.6 to 1 doubling per day (Methods). After each cycle of random 50% mortality in this simulation, the population is replenished in proportion to both the relative abundance of remaining individuals and their assigned species-specific division rates (Methods). The outcome of this model is ecological drift (Fig. 2a) with directed community evolution where species richness declines toward a steady-state diversity (Fig. 2b) within a narrowing range of division rates (Fig. 2c). A critical difference between this simulation and our purely stochastic model (Fig. 1) is that, in the case of trophic exclusion, selection against slower division rates (species) occurs uniformly across spatial scales. In other words, while physical mixing of populations over increasing distance with time effectively eliminates stochastic exclusion as a significant factor constraining phytoplankton diversity (see above), it does not have a similar impact on trophic exclusion. What the combination of physical mixing, random mortality, and proportional repopulation does in the trophic exclusion scenario is ensure that all species within the model that fall within the selected range of division rates are retained in the metacommunity. In other words, trophic exclusion narrows the diversity of extant species in a given environment until similarities in fitness result in stochastic processes becoming dominant.

A key difference between resource-based competitive exclusion and the trophic exclusion described above is that the former “bottom-up” process focuses on differences between species in terms of resource acquisition, whereas trophic exclusion focuses on a given species’ growth-loss balance (*r*′) relative to that of the entire phytoplankton community within its size class. For the simulation shown in Fig. 2, all individuals are assumed to have the same probability of mortality per generation and *r*′ for a given species is determined by its assigned division rate. Alternatively, an equivalent result can be achieved by assuming equal division rates for all species and making loss rates species-specific. Thus, adaptations reducing mortality, for example through morphological or chemical grazing deterrents or protection from viral infection, can be equally effective as those targeting enhanced resource acquisition (i.e., division rate) in terms of ensuring a given species’ retention in the community. In addition, contrasting adaptive strategies can cause the relative position of a given species’ fitness in the community to change as growth conditions vary (e.g., adaptations to low-light conditions will be advantageous at some times and disadvantageous at others). Since the time-scale for species loss increases (even exceeding the annual cycle) as the range in *r*′ narrows with trophic exclusion (Fig. 2b), the effect of environmental variability (“disturbance”) implies that all species with similar time-averaged *r*′ values ($\overline {r^\prime }$) can coexist (Supplementary Fig. 1).

Trophic exclusion has an additional important attribute: it independently regulates diversity across size classes. Specifically, while some grazers feed wholesale across the phytoplankton size domain (e.g., gelatinous tunicates feeding with mucous webs [22, 23]), the absolute size range grazed upon by herbivores is generally proportional to their average prey size [12, 24–29]. This phenomenon plays a decisive role in controlling phytoplankton size distributions [12] and it implies that trophic exclusion functions within, not between, feeding size ranges. In other words, $\overline {r^\prime }$ distributions will be similarly narrowed for all size classes within a given number of generations (albeit over a longer absolute time for lower $\overline {r^\prime }$ values), but different median $\overline {r^\prime }$ values for these distributions can be sustained between size classes. Thus, coexistence of small rapidly-growing species and large slowly-growing species is easily permissible under trophic exclusion, even in temporally-stable and low-nutrient aquatic environments such as the central ocean gyres.

### Size-dependent diversity

**Fig. 3:**
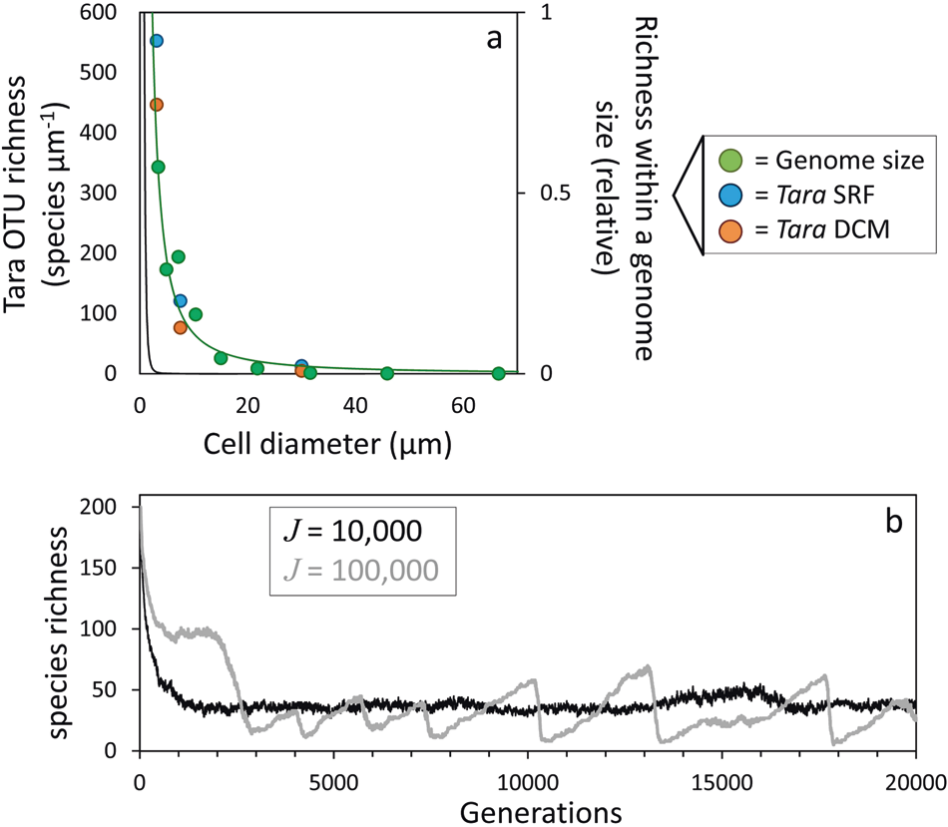
Size dependence of phytoplankton biodiversity. **a** green symbols, right axis = relative genome-size abundance for eukaryotic cells as a function of cell diameter (Methods). Green line = power function fit to genome-size data (slope = −1.5). Blue and orange symbols, left axis = richness of operational taxonomic units (OTUs) per unit cell diameter for the surface (SRF, blue) and deep chlorophyll maximum (DCM, orange) base on the three cell-size classes measured during *Tara* Oceans. Solid black line = anticipated phytoplankton diversity based on neutral theory and the canonical phytoplankton size distribution of −4 log–log slope [12]. **b** Variation in phytoplankton diversity for 200 modeled species in populations of 10,000 and 100,000 individuals following ecological drift and trophic exclusion with fixed probabilities of neutral and beneficial speciation (Methods).

In neutral theory, diversity of a metacommunity is a function of speciation rate per birth, *υ*, and the total number of individuals in the community (Eq. 1) [14]. Empirical tests of this prediction in terrestrial systems have been inconclusive [30], but for natural phytoplankton communities the ramification is extreme. Specifically, the abundance versus size (cell diameter) distribution of phytoplankton typically exhibits a log–log slope of −4, meaning that for every 20 µm diameter cell there are ~1000 cells of 3 µm diameter and ~100,000 cells of 1 µm diameter [12]. Equation 1 accordingly predicts the seemingly unlikely equivalent size distribution for species diversity (Fig. 3a; black line).

Speciation is generally not a point event, but rather a protracted process that requires acquisition of (a) new coding gene(s) and persistence of this acquisition within a lineage for sufficient time for reproductive barriers to emerge [31]. Interestingly, the amount of coding DNA is conserved at ~10^4^ genes across eukaryotic genomes [32]. If the rate of gene mutation is proportional to coding gene number, then this observation implies that *υ* will be, to first order, size-independent within the phytoplankton [33–35, but see 36]. With respect to fitness, acquisition of a new gene can have three possible fates in terms of trophic exclusion. It can be associated with a decrease in $\overline {r^\prime }$ and thus will be removed from the population. It can have no impact on $\overline {r^\prime }$ and thus will add to diversity. Or, it may slightly enhance $\overline {r^\prime }$, in which case it will diminish diversity by leading to the loss of other species. The reason for this latter outcome is that introduction of an enhanced fitness level will cause trophic exclusion processes to up-shift the $\overline {r^\prime }$ distribution and eliminate previously successful species on the downside of this distribution. Thus, biodiversity of the phytoplankton depends on the balance between neutral speciation rates (*υ*_*N*_) with respect to $\overline {r^\prime }$ and beneficial rates (*υ*_*B*_).

The influence of neutral and beneficial speciations on diversity can be illustrated by modifying our stochastic model with trophic exclusion (Fig. 2) such that constant values of *υ*_*N*_ and *υ*_*B*_ are applied to populations of 10,000 and 100,000 individuals (Methods). The outcome of this simulation is that both populations have similar mean diversities, but fluctuations in diversification and extinction increase (i.e., mean species longevity decreases) with increasing population size (Fig. 3b). In other words, our prediction is that, due to trophic exclusion processes, sustained diversity across the phytoplankton size domain does not follow the neutral theory expectation of *θ* $\propto$ *J υ*, but rather is independent of *J*. However, as discussed below, diversity may still exhibit significant size-structuring if other independent mechanisms exists.

The total DNA content of algal species varies by over 5 orders of magnitude (the full range across the eukaryotic domain exceeds 6 orders of magnitude) [37]. This tremendous range in genome size is largely driven by random insertions and deletions of noncoding DNA (*nc*DNA) that evolve in a manner proportional to genome size [38]. Thus, the a priori expectation is for many more small than large genomes, irrespective of any phenotypic selection for or against genome size [38]. The same size-dependent distribution in eukaryotic phytoplankton diversity (Fig. 3a; green symbols) can also be expected because cell volume is highly correlated (*r* = 0.96) in a near 1:1 manner (slope = 0.97) with genome-size [39]. Thus, although the interplay between *υ*_*N*_ and *υ*_*B*_ under trophic exclusion minimizes the dependence of diversity on metacommunity size (thus cell size) (Fig. 3b, Supplementary Fig. 2), we still predict a size dependence in phytoplankton diversity because proportional genome-size evolution driven by *nc*DNA redistributes species in a manner favoring smaller cells. This drift in species size carries with it the $\overline {r^\prime }$-relevant phenotypic implications of size for single-celled organisms (e.g., nutrient acquisition, division rate, and loss rate [37, 38, 40]), yielding a rate of genome-size evolution in these single-celled eukaryotes that is essentially indistinguishable from multicellular organisms where body size and cell size (i.e., genome size) are independent [36].

### Insights from the sea

**Fig. 4:**
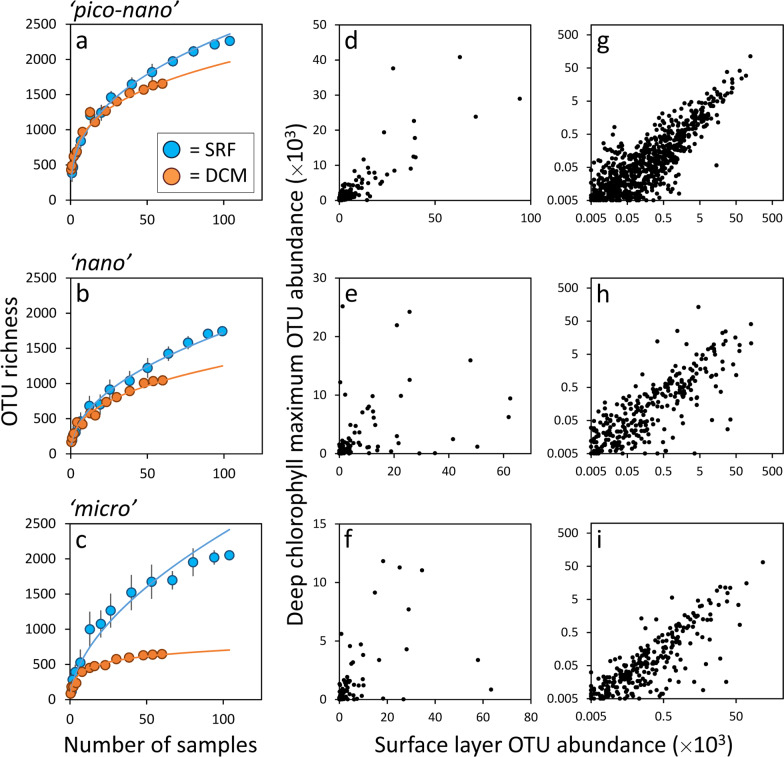
Size-dependent phytoplankton biodiversity observed during *Tara* Oceans. Top, middle, and bottom panels correspond to “pico–nano”, “nano”, and “micro” size class, respectively. **a**–**c** Cumulative phytoplankton operational taxonomic unit (OTU) richness as a function of number of samples analyzed (each sample corresponds to a unique geographic location) (Methods). Blue symbols = surface (SRF) samples. Orange symbols = deep chlorophyll maximum (DCM) samples. Colored solid lines are power function fit to data except for the orange line in (**c**), which is better fit by a logarithmic function. Vertical bars show standard deviations for 20 Monte Carlo sample aggregations (Methods). **d**–**i** Comparison of species’ abundances in the SRF and DCM for the three size classes and all *Tara* Oceans samples. Data are for species with at least five observed individuals in the DCM. **d**–**f** Data plotted on normal axes, but truncated to eliminate extremely abundance species to better illustrate all other species. **g**–**i** Data for all species plotted on log-transformed axes (i.e., no truncation of most abundant species).

*Tara* Oceans was a circumglobal expedition during which extensive samples were collected for sequencing of eukaryotic plankton DNA for the V9 region of the 18S rRNA gene [2]. Strict bioinformatics quality control was applied to these sequences and distinct metabarcodes have been clustered into biologically meaningful operational taxonomic units (OTUs) [41]. We investigated patterns of eukaryotic phytoplankton biodiversity based on these OTUs for “pico–nano” (0.8–5 µm), “nano” (5–20 µm), and “micro” (20–180 µm) size classes from surface (SRF) and deep chlorophyll maximum (DCM) samples (Methods). Total SRF diversity is similar for all three size classes (Fig. 4a–c; blue symbols), although the non-saturating relationship between OTU richness and sample number for each category suggests that significant eukaryotic phytoplankton diversity is yet to be discovered. Size ranges encompassed within the “pico–nano” to “micro” classes increase roughly exponentially. Accordingly, phytoplankton diversity per unit cell diameter decreases as a power function with a slope slightly steeper than −1 (Fig. 3a; blue symbols). This relationship is not consistent with diversity varying as a function of population size, *J* (Eq. 1), and is instead remarkably similar to our prediction based on trophic exclusion principles and genome-size evolution (Fig. 3a).

Phytoplankton diversity (richness) in the DCM decreases and exhibits a greater degree of saturation with increasing cell size compared to the SRF, but retains a similar power function relationship with cell diameter (Figs. 4a–c, 3a; orange symbols). In general, the DCM is characterized as a relatively stable, energy-limited, and nutrient-enriched environment that contrasts starkly with the physically-dynamic, high-light, and low-nutrient conditions of surface mixed layers at low- to mid-latitudes. A stark difference in species composition might therefore be expected between these environments, yet we find that across the three size classes ≥98% of all DCM species with at least five observed individuals are also found in the SRF. Nearly all species unique to either the DCM or SRF are very rare (Supplementary Fig. 3), suggesting their absence at a given depth is as likely a reflection of insufficient sampling as it is of specific environmental adaptions. Furthermore, we find that “pico–nano” species exhibit a similar level of dominance in both the SRF and DCM (Fig. 4d, g). This correspondence likewise applies to first order in the “nano” and “micro” size classes (Fig. 4h, i), but here significant shifts in species dominance are also seen between SRF and DCM communities (Fig. 4e, f) (in other words, some dominant species in the DCM are present at lower relative abundances in the SRF, and vice versa). This finding suggests stronger environmental influences on community structuring in these larger size classes.

Despite the severe contrast of SRF and DCM growth conditions, the remarkably conserved richness of “pico–nano” diversity between depth horizons suggests little latitude for physiological invention in this smallest size class, where the slight reduction in diversity at the DCM possibly reflects a stronger impact of trophic exclusion in a more temporally-stable environment. For the two larger size classes, the contrast of SRF and DCM communities does not reflect a proliferation of different species between horizons so much as a strong “down-selection” in the DCM of the richer SRF diversity. This observation may imply that increasing cell size allows for a greater multiplicity of physiological and morphological adaptations to achieve a given trophically-selected $\overline {r^\prime }$ in the physically-dynamic SRF environment, but a stronger basis for exclusion in the more stable DCM. Irrespective of such mechanistic interpretations, the *Tara* Oceans data most importantly suggest a relatively weak role of these environmental niches on species selection at the level defining OTUs, which we here propose is in part due to the predominantly asexual nature of phytoplankton reproduction.

As noted above, the path to speciation following acquisition of beneficial new genes requires the establishment of barriers to genome alignment during meiosis. Asexual reproduction bypasses such barriers and provides many generations for successful new physiological inventions to propagate within a metacommunity. Accordingly, we suggest that promiscuous asexual reproduction engenders enhanced phenotypic diversity that is carried within a population as small genomic variations that rarely lead to differences that exceed the homology criteria used to define OTUs. An example of such subspecies phenotypic variability is beautifully displayed in the most common (prokaryotic) phytoplankton of the global ocean, *Prochlorococcus*. Variants of this species express a phylogenomic hierarchy of adaptations for phosphate, nitrogen, and/or iron uptake and assimilation [41–43]. When a particular nutrient is replete, *Prochlorococcus* variants with the simplest genome are selected, but as a nutrient becomes more scarce populations become dominated by variants with genomes that expand the diversity of utilizable substrates for the limiting resource [41, 44]. Intraspecific physiological variability is likewise observed in eukaryotic phytoplankton [45].

The notion that the relative frequency of asexual versus sexual reproduction plays a governing role in the balance between sustained phenotypic variation within a species and emergence of new species has implications regarding speciation across phytoplankton lineages. Specifically, diatoms are unique in being the only phytoplankton to produce a continuous siliceous cell wall. Asexual reproduction in diatoms causes the average size of this ‘frustule’ to decrease in a population over time and, in most species, size-reconstitution requires sexual reproduction. This enhanced requirement for sex (along with the diplontic genome of diatoms) imparts a higher likelihood for reproductive barriers to emerge between variants and thus results in an increased tempo of speciation relative to other largely haploid and asexually reproducing algal lines [46]. This prediction is consistent with genome-based reconstructions of phytoplankton diversification [38, 47].

### Synthesis

Biodiversity is a manifestation of the balancing point of speciation and extinction within a metacommunity. Darwin [48] envisioned this balance as guided by niche-differentiated resource-based competitive exclusion. This philosophy remains deeply embedded in current ecological thinking and was the basis for Hutchinson’s Paradox of the Plankton [1, 6]. Fundamentals of competitive exclusion are expressed in modern aquatic ecosystem models, where phytoplankton are treated as a diffuse field defined by an elemental stock (e.g., N or C) and growth-limiting resources are a commodity uniformly accessible across phytoplankton groups [49, 50]. In this construct, nutrient uptake traits govern community structuring so strongly that modeled populations must often be “reseeded” with taxa to counteract severe resource-based competitive exclusion [7, 51]. Such a stark contrast between model behavior and natural populations is a harbinger of something amiss and consideration of body-length spacing between phytoplankton suggests that the culprit is the presumption in models of direct resource competition between individuals and size classes [10–12].

In the absence of direct competition, random speciation and mortality are still anticipated to yield a steady-state diversity, but one that follows a random ecological drift. This dynamic of biodiversity is the focus of neutral theory, which is often viewed as the “null model” against which additional complexities must be tested [9, 52]. For large-bodied terrestrial organisms within a given trophic level, application of reasonable speciation rates in neutral theory to appropriate metacommunity numerical abundances yields predictions of biodiversity that are broadly consistent with observations [14], but is this success mechanistic or fortuitous?

Application of neutral theory to the extreme size distribution and overall abundance of phytoplankton results in grossly inaccurate predictions of biodiversity. Such drastic inconsistencies motivate reinterpretation. Here, we convey the growth environment of phytoplankton as a competition-neutral resource landscape where physical mixing and stochastic processes sustain biodiversity, but where predator-prey interactions direct the ecological drift in diversity through selection of species by virtue of adaptations impacting their time-averaged balance between division and loss rates ($\overline {r^\prime }$). This latter trophic exclusion process, along with influences of genome-size evolution, the interplay of neutral and beneficial speciation events, and environmental variability, yields the expectation of a size-dependent phytoplankton diversity far richer than implied by readily-identifiable environmental niches of the pelagic environment (i.e., Hutchinson’s Paradox of the Plankton), but also far more constrained than allowed by neutral theory [14].

Neutral theory begins with the assumption that individuals within a community do not compete, leaving stochastic processes to explain biodiversity. Our findings suggest that ecological processes create communities of equally fit species in terms of time-averaged $\overline {r^\prime }$ and that, as these processes constrict the $\overline {r^\prime }$ distribution, stochastic behavior becomes increasingly important in governing temporal dynamics of diversity. Selection for a community of equally fit species is very different than the neutral theory concept of individuals in a community not competing. Thus, in answer to the “mechanistic or fortuitous” question posed above, we suggest that successes of neutral theory reflect both. They are fortuitous because the theory is tested using data from natural communities where nonrandom processes have already selected for equally competitive species in terms of their time-averaged $\overline {r^\prime }$. They are mechanistic because the remaining dynamics of biodiversity in these selected communities is dominated by the stochastic processes captured in neutral theory. These insights can be concealed by the smaller population sizes and slower turnover of the larger-bodied terrestrial organisms to which neutral theory is most often applied, but they emerge more clearly when the theory is applied to the astronomical numbers and rapid turnover of the plankton.

The true complexity of ecological interactions in planktonic systems will forever elude their complete encapsulation in models. The extreme simplicity of the models employed here represents the other end member, but their intention is for illustration of basic processes rather than a full accounting of natural complexity. Missing from our models are many ecological details, such as the role of species co-dependencies on $\overline {r^\prime }$ and the influence of selective versus untargeted loss processes. Inclusion of such details will influence modeled steady-state diversity and the potential for stochastic exclusion of rare species, which can persist in microbial metacommunities [53]. Our model also does not explicitly include processes of physical mixing, ocean circulation, or seasonal environmental variability. What we do find is that the time-scale of species selection under trophic exclusion decreases as the distribution of $\overline {r^\prime }$ narrows, suggesting observed diversity at a given location will often contain a signature of previous selections from disparate growth environments brought together by physical transport. With respect to seasonal variability (“disturbance”), the mechanisms of trophic exclusion might, on one hand, imply enhanced diversity in more dynamic high-latitude systems due to stronger temporal shifts in species’ relative positions within the *r*′ distribution (much like the enhanced *Tara* SRF diversity compared to the DCM (Fig. 4a–c)). On the other hand, extreme environments might be associated with reduced diversity if radical adaptations to these conditions are found amongst a limited number of species creating the upper tail of the *r*′ distribution selected upon by trophic exclusion (such as suggested by the decrease in diversity with increasing cell size in the *Tara* DCM data (Fig. 4a–c)). Seasonal successions in species dominance in such environments has already been linked to such physiological adaptations influencing *r*′ [12, 46].

Biodiversity is inevitably influenced by stochastic processes, yet alignment of species biogeography with physiological adaptations attests to selective pressures associated with, at the very least, broad environmental niche differentiation. Within each niche, trophically-directed ecological drift sustains diversity richer than expected under resource-based competitive exclusion but also constrained relative to neutral theory. Diversity of classically-defined species (i.e., individuals capable of interbreeding) is also inherently lessened in asexually reproducing microbes, such as phytoplankton, simply due to relaxation of reproductive barriers that otherwise lead to speciation, creating populations of phenotypic variants that muddle the very concept of a species. While *Tara* data provided insight here on OTU-based biodiversity for the broadly defined metacommunity of the mid- and lower-latitude global ocean, the rapidly-growing body of genome sequencing data should allow similar but more regionally-constrained analyses, as well as more detailed analyses of subspecies diversity.

We propose that trophic exclusion, stochastic processes, genome-size evolution, and implications of asexual reproduction on speciation are all elements defining the richness of phytoplankton communities within an environmental niche and that the explanation for the ‘Inverted Paradox of the Phytoplankton’ as to why pelagic diversity is impoverished compared to plants, insects, and other terrestrial organisms ironically lies in the original issue troubling Evelyn Hutchinson: phytoplankton diversity is low because the pelagic environment presents a comparative paucity of significant environmental niches.

## Methods

### Modeling software and access to code

The model runs described below were all executed using MATLAB Release R2020b (The MathWorks, Inc, Natick, Massachusetts, United States, https://www.mathworks.com). Matlab scripts used to generate results shown in Figs. 1, 2, 3 are available through GitHub (O’Malley, R (2021) Neutral_new [Source code]. https://github.com/RTOMalley/Neutral_new).

### Stochastic model

Results shown in Fig. 1 are for a purely stochastic model of diversity executed for three populations of 10,000, 100,000, and 1,000,000 individuals where each population is initiated with 10,000 species, all of which have identical division rates. In this model, every individual reproduces with each generation such that the total population size doubles. Following division, 50% of the individuals are randomly removed, representing the required loss rate of a steady-state system with respect to population size. In the absence of immigration, this simulation results in an ecological drift in diversity that eventually leads to a single remaining species. This ‘baseline’ simulation is then modified to include an immigration rate (*υ*) of either 0.03% or 0.3% per generation. Immigrating individuals are randomly assigned a species designation drawn from the initial pool of 10,000 species.

### Stochastic exclusion in the ocean

Results from the model simulation in Fig. 1 with no immigration show that, for a given diversity, the time required for significant stochastic exclusion to ensue scales with population size. For example, a 5% decrease in diversity is exceeded within the first generation of the 10,000 individual simulation, but requires 10 and 100 generations for the populations of 100,000, and 1,000,000 individuals, respectively. Within a physically-homogenized water mass 1 m deep and 1000 m wide with a phytoplankton concentration of 1 or 10^5^ individuals per milliliter, the total number of cells is 10^12^ and 10^17^, respectively. For these population sizes and assuming an extant diversity of 10,000 species, a 5% reduction in diversity from pure stochastic exclusion requires 10^8^ to 10^13^ generations, or 10^5^ to 10^10^ years assuming one division per day. If we consider the volume of water physically homogenized within even a single year, it is clear that simple stochastic exclusion alone places no constraints on phytoplankton diversity.

### Stochastic model with trophic exclusion

Results shown in Fig. 2 are for a modified version of the stochastic model used to generate Fig. 1. For the trophic exclusion simulation, we assumed 200 initial species within a population of 10,000 individuals (i.e., 50 individuals per species), which is the midpoint of the range used to initiate the purely stochastic simulations in Fig. 1 (i.e., 1 to 100 individuals per species). Each species in the trophic exclusion simulation is assigned one of ten fixed division rates that range from 0.6 to 1.0 division per day. For each day of the simulation, the total abundance of each species first increases following its assigned division rate and then 10,000 individuals from the resultant population are randomly selected as having survived predation. In this simulation, trophic exclusion progressively selects for species with higher division rates such that the distribution of division rates narrows (Fig. 2c). However, there is also a purely stochastic element to this model that decreases diversity (just as in the model for Fig. 1). Thus, for neighboring water parcels, trophic exclusion processes equally narrow distributions in division rates, but stochastic processes randomly determine which species within this distribution remain. In such a case, species diversity available for immigration decreases in time. To simulate this effect, we assumed a 0.3% immigration between generations, but the species assigned to this immigration are randomly selected from any of the initial 200 species that possess a division rate within the narrowing distribution of division rates resulting from trophic exclusion. This stochastic model with trophic exclusion was executed three times for 3650 generations, with each model run yielding qualitatively similar results (the stochastic element of the model results in the retention of different species and different relative dominances). Data shown in Fig. 2a, b are from one of these three model runs. Results shown in Fig. 2c represent the average of all three runs.

### Stochastic model with trophic exclusion and speciation

Results shown in Fig. 3b are for the stochastic model with trophic exclusion and immigration, but also with the addition of new species resulting from acquisition of neutral and beneficial phenologies (i.e., depicting true speciations within the metacommunity). For these simulations, the model was initiated as described above with an immigration rate of 0.03% per generation and 200 species growing at rates between 0.6 and 1 division per day. Two population sizes of 10,000 and 100,000 individuals were modeled and simulations were extended to 20,000 generations. A probability of 0.00002% was assumed for neutral (*υ*_*N*_) and beneficial (*υ*_*B*_) speciation, thus ten-times more new species emerged over time in the population of 100,000 than in the population of 10,000 individuals. When a new neutral species was introduced, it was assigned a division rate randomly selected from division rates extant within the existing population. When a new beneficial species was introduced, it was assigned a division rate 0.001 divisions per day greater than the maximum division rate within the existing population. We note here that the intent of these simulations is to illustrate the basic influence of *υ*_*N*_ and *υ*_*B*_ in balancing sustained species diversity within a computationally reasonable number of population generations (20,000 generations in Fig. 3b). Actual speciation rates in nature are far lower than those applied in our model.

### Genome size and Tara Oceans data

We extracted the size distribution of eukaryotic genomes from Fig. 6b of Oliver et al. [38] and converted the reported base pairs (bp) into picograms (pg DNA) as: pg DNA = bp/(0.978 H 10^9^) (http://www.genomesize.com/faq.php). Phytoplankton cell volume (CV) was then calculated from pg DNA following Shuter et al. [39]: CV = e^6.9 + 0.97 ln(pg DNA)^. Cell diameter was estimated from CV assuming spherical cells.

To investigate phytoplankton species richness and abundance in natural populations, we analyzed the uniformly-collected *Tara* Oceans 18S rRNA gene amplicon sequencing data. OTU were derived from the V9 region (provided to us as tabulated data by Dr. de Vargas’ lab in Station Biologique de Roscoff). Full descriptions of the DNA sequencing and read processing are given in [2, 40, 54]. Our analysis was intended to evaluate phytoplankton diversity within a broadly-consistent environmental domain (i.e., warmer waters that are permanently stratified within the euphotic layer and only modestly seasonal), so we limited our analysis to samples collected at 124 stations located primarily in tropical and subtropical waters (i.e., arctic samples were excluded) [2]. Samples from the surface (SRF) and DCM were separated by sequential filtrations into “pico–nano” (0.8–5 µm), “nano” (5–20 µm), and “micro” (20–180 µm) size classes (note, a forth size class of 180–2000 µm was also collected that contains many endosymbiotic species, but this size class was not included in our analyses). Because filtration-based size fractionation is imperfect (i.e., cells of a given physical size can be captured on filters with larger pore sizes), we assigned a single size class to each OTU based on which group (pico–nano, nano, or micro) it was most abundant. To construct the diversity versus sample number plots, we conducted a Monte Carlo random sampling from all available samples to remove any geographic dependence of species distributions. This Monte Carlo sampling was repeated 20 times for each depth and size class, with the average of all 20 runs reported in Fig. 4a–c. The total number of samples analyzed for a given depth differed between size classes (99 to 117 for the SRF, 53 to 60 for the DCM). To calculate diversity per unit cell size, we therefore extrapolated diversity for each size class to the maximum number of samples collected at a given sampling depth (i.e., SRF = 117, DCM = 60) based on the final slope of the relationships between sample number and diversity shown in Fig. 4a–c. Total OTUs for each size bin and depth were then divided by the size range encompassed within a given class to determine the mean number of OTUs per unit size. The centroid diameter for the nano and micro size classes was calculated assuming a phytoplankton size distribution with a log–log slope of −4 [12]. For eukaryotic pico–nano sized phytoplankton, the size distribution slope of −4 is not expected to continue between 0.8 and 5 µm (as the lower end of this range becomes dominated by prokaryotic species). The centroid diameter for the pico–nano size class was therefore determined from flow cytometry data collected in oligotrophic waters during the North Atlantic Aerosol and Marine Ecosystem (NAAMES) study [55], which yielded centroids ranging from 2.8 to 3.8 µm (median = 3.1 µm).

## Supplementary information

Supplementary figures
