# Supplementary material for: Phytoplankton biodiversity and the inverted paradox
Source: ISME Commun. 2021 Oct 6;1:52. doi: 10.1038/s43705-021-00056-6 (PMC9723737; doi:10.1038/s43705-021-00056-6)

This file contains supplementary figures for the manuscript, "*Phytoplankton Biodiversity and the Inverted Paradox*", by Behrenfeld *et al*.

Supplementary Figure S1. **Phytoplankton biodiversity under directed ecological drift from trophic exclusion with seasonally-varying division rates.** In figure 2 and the 'Trophic Exclusion' subsection of the main manuscript, we discuss results for our stochastic model where the trophic exclusion process is illustrated for a population of 10,000 individuals consisting of 200 species and where each species is assigned a fixed division rate (μ) ranging from 0.6 to 1 doubling per day. In this scenario, the probability of mortality per generation is uniform between species, so r' is determined by μ, but we note in the manuscript that adaptations reducing species-specific mortality can be equally effective as those targeting enhanced μ in terms of ensuring a given species' retention in the community. We then suggest that contrasting adaptive strategies cause the relative position of a given species' fitness in a community to vary as growth conditions change and, consequently, that all species with similar time-averaged *r'* values ($\overline{r'}$) should be retained. To evaluate this latter conclusion, we executed additional runs of the stochastic model with trophic exclusion where each of the 200 species had an annual mean μ of 0.8 doubling per day but where each species was assigned a unique annual cycle in μ. These species-specific annual μ cycles had amplitudes ranging from 0 to ±0.2 doubling per day (thus encompassing the full range of μ in the original simulation) and (for amplitudes > 0 doubling per day) phases that peaked on different calendar days for the different species. Because the probability of a given species being removed from the community at the beginning of the simulation depended on the position of its annual μ cycle relative to the start date of the simulation, paired model runs were executed with Julian start dates of 1 and 180 and average species abundances calculated for the two runs. These paired runs were repeated 10 times and yielded similar results. An example outcome of the simulation is provided below, showing that nearly all of the initial 200 species are retained in the modeled community for the duration of the simulation because of their equivalent time-averaged $\overline{r'}$ values.

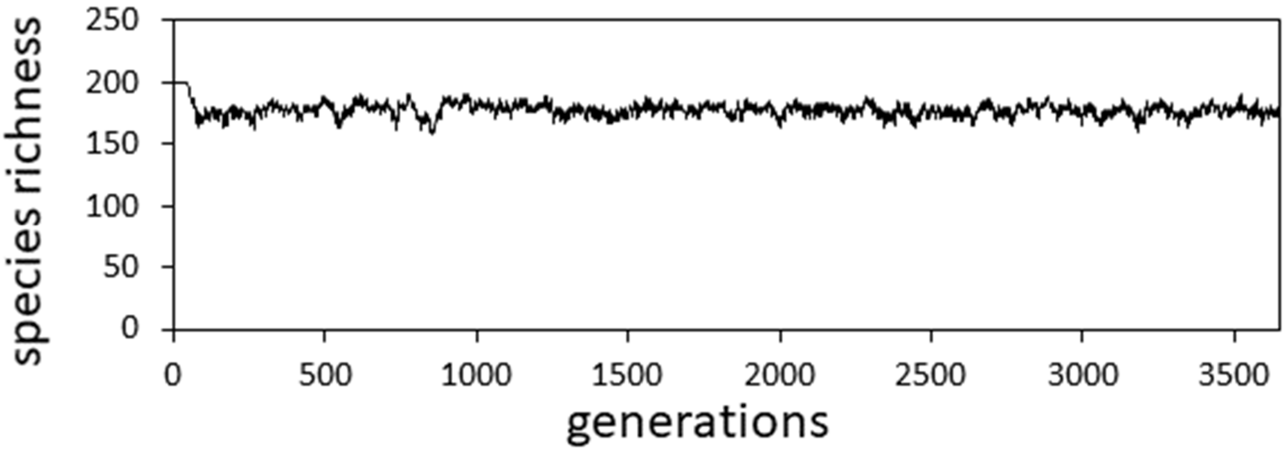

Supplementary Figure S2. **Phytoplankton biodiversity for multiple runs of the stochastic model with trophic exclusion and speciation**. Variation in phytoplankton diversity for 200 modeled species in populations of (black lines) 10,000 and (gray lines) 100,000 individuals following ecological drift and trophic exclusion with fixed probabilities of neutral and beneficial speciation. Results are shown for four independent model runs for each population.

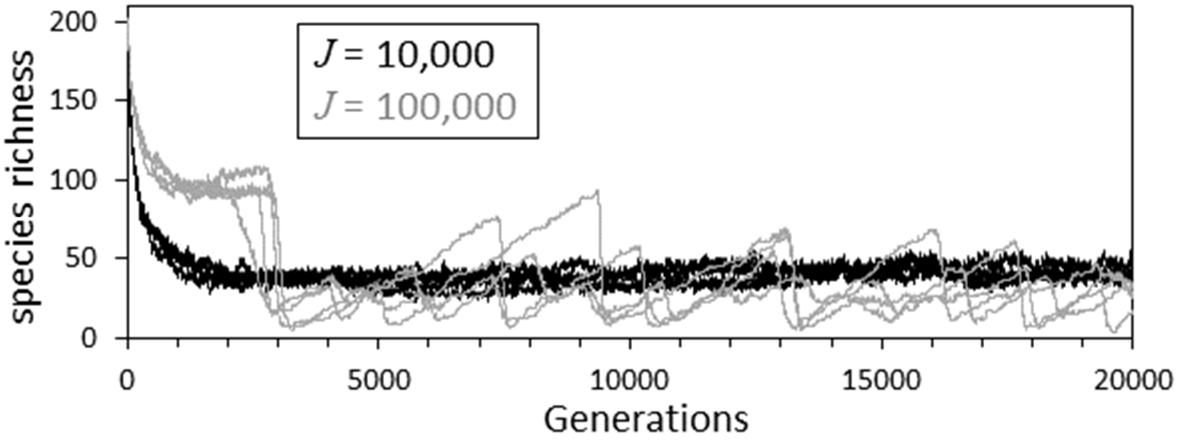

Supplementary Figure S3. **Rank-abundance relationships for size-dependent phytoplankton biodiversity observed during *Tara* Oceans.** Top, middle, and bottom panels correspond to ‘pico-nano’, ‘nano’, and ‘micro’ size class, respectively. Black symbols = OTUs common to both the surface (SRF) and deep chlorophyll maximum (DCM). Red circles = OTUs unique to the SRF. Blue circles = OTUs unique to the DCM. OTUs are ranked along the x-axes from the most (left) to least (right) abundant based on total number of individuals observed in both the SRF and DCM.

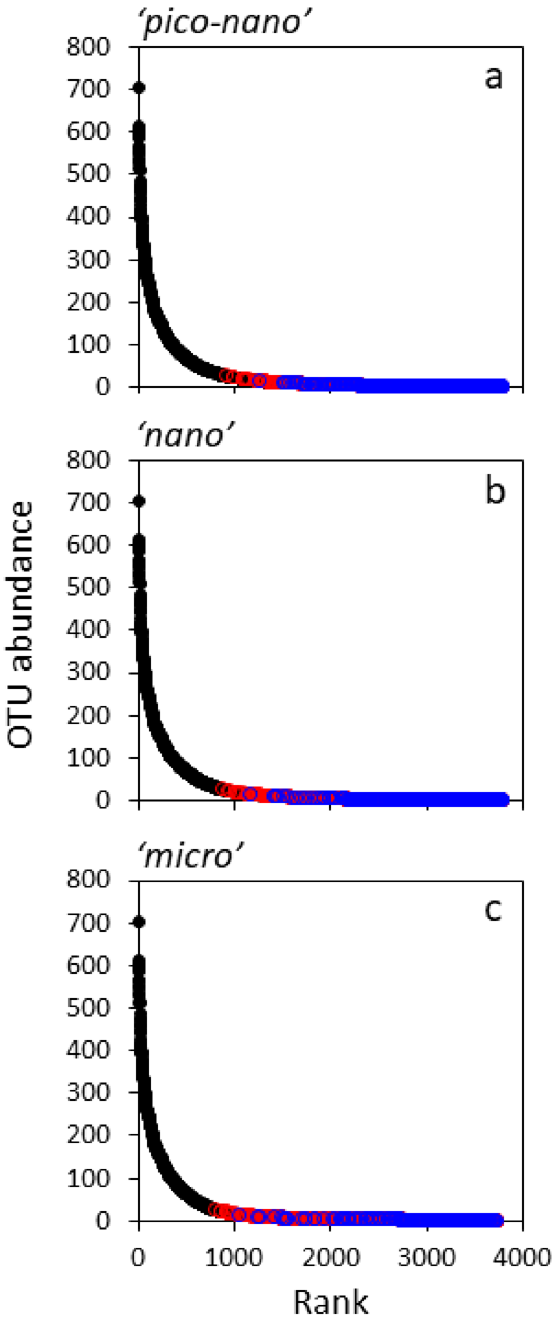

Supplement: Supplementary file 1 — Supplementary figures [file 43705_2021_56_MOESM1_ESM.pdf]
